# Identification of GABA receptors in chick cornea

**Published:** 2012-05-01

**Authors:** Zhen-Ying Cheng, Mary Chebib, Katrina L. Schmid

**Affiliations:** 1 Myopia Center, Department of Ophthalmology, Qilu Hospital, Shandong University, Jinan, Shandong, China; 2 Faculty of Pharmacy, University of Sydney, Sydney, New South Wales, Australia; 3 School of Optometry and Vision Science, Faculty of Health, and Vision Improvement Domain, Institute of Health and Biomedical Innovation, Queensland University of Technology, Brisbane, Australia

## Abstract

**Purpose:**

The cornea has an important role in vision, is highly innervated and many neurotransmitter receptors are present, e.g., muscarine, melatonin, and dopamine receptors. γ-aminobutyric acid (GABA) is the most important inhibitory neurotransmitter in the retina and central nervous system, but it is unknown whether GABA receptors are present in cornea. The aim of this study was to determine if GABA receptors are located in chick cornea.

**Methods:**

Corneal tissues were collected from 25, 12-day-old chicks. Real time PCR, western blot, and immunohistochemistry were used to determine whether alpha_1_ GABA_A_, GABA_B_, and rho_1_ GABA_C_ receptors were expressed and located in chick cornea.

**Results:**

Corneal tissue was positive for alpha_1_ GABA_A_ and rho_1_ GABA_C_ receptor mRNA (PCR) and protein (western blot) expression but was negative for GABA_B_ receptor mRNA and protein. Alpha_1_ GABA_A_ and rho_1_ GABA_C_ receptor protein labeling was observed in the corneal epithelium using immunohistochemistry.

**Conclusions:**

These investigations clearly show that chick cornea possesses alpha_1_ GABA_A_, and rho_1_ GABA_C_ receptors, but not GABA_B_ receptors. The purpose of the alpha_1_ GABA_A_ and rho_1_ GABA_C_ receptors in cornea is a fascinating unexplored question.

## Introduction

The cornea, as the eye’s first refracting surface, plays an important function in vision, is highly innervated and has precise mechanisms for maintaining optical clarity. The chick cornea has been suggested to be an excellent model for the study of wound healing, scar tissue formation and neuronal re-innervation [1] and thus it is important to learn more about chick corneal anatomy and physiology. Many neurotransmitter receptors have been shown to be present in the cornea, e.g., muscarinic [2], melatonin [3], and dopamine receptors [4], but others appear to have not been studied to any siginificant degree (e.g., γ-aminobutyric acid [GABA] receptors). In the literature there is a sole report of GABA receptor expression in cultured human stem cells [5].

GABA is the major inhibitory neurotransmitter of the retina and central nervous system. It exerts its effects through three classes of membrane receptors, GABA_A,_ GABA_B_, and GABA_C_ which contain multiple sub-units [6-14]. These include 16 subunits (alpha1–6, beta1–3, gamma1–3, delta, epsilon, theta, and pi) combined as GABA_A_, and 3 rho subunits (rho1–3), which form homomeric receptors composed of rho subunits, and are commonly called GABA_C_ receptors. GABA_A_ and GABA_C_ are ionotropic receptors involving chloride channels that mediate fast synaptic inhibition when activated by GABA [13]. GABA_B_ receptors are members of the metabotropic receptor family that via G proteins interact with neuronal inwardly rectifying potassium and voltage-gated calcium channels and when activated mediate slow synaptic inhibition [14].

Knowledge regarding the distribution of GABA receptors in the eye is very limited and information on GABA receptors in the cornea even more sparse. GABA_A_ [6-8], GABA_B_ [9-11], and GABA_C_ [12] receptors have been reported in the retina. We have recently shown that rho_1_ GABA_C_ receptors are present in chick sclera [15]. A small number of cultured human corneal stem cells have been shown to exhibit GABA_A_ receptor immunoreactivity [5]. We found no reports of studies of GABA receptor expression in any animal species, although there is a report of GABA modification of the rabbit corneal endothelial fluid pump [16] and an observation of activity of the GABA-synthesizing enzyme, glutamic acid decarboxylase (GAD), in rat cornea [17].

A range of other neurotransmitter receptors have been reported in corneal tissues of a diverse animal species [2-4,18] and human tissue [19,20]. The muscarinic receptor subtypes M1 and M2 are expressed in bovine corneal epithelial cells [2]. In the rabbit cornea, D1 and D2 dopamine receptors have been localized to both epithelial and endothelial layers [4]. In the mouse cornea, retinoic acid (RA) receptors have been detected in the epithelium and stroma [18]. In the *Xenopus laevis* eye, melatonin receptors have been visualized in the corneal epithelium, fibroblasts, and endothelium [3]. In the human cornea, muscarinic M1–5 receptors have been observed on corneal epithelial cells [19], and M2, M4, and M5 are expressed in corneal endothelium [20].

Although GABA receptors are located primarily in retina and the central nervous system, reports have shown the presence of GABA receptors in non-neural tissues [21-36]. For example, GABA_A_ receptors have been shown to be located in murine gut [21], in cat carotid body [22], in rat taste bud [23], in human thyroid [24], human hepatocellular [25], human peripheral blood mononuclear cells [26], and human prostate [27]. GABA_B_ receptors have been reported in spider leg mechanosensilla [28], rat testis and sperm [29], the rat olfactory bulb [30], the rat taste bud [23], the rat gastrointestinal tract [31], and rat cardiomyocytes [32]. In human, GABA_B_ receptors are reported in airway epithelium [33] and fallopian tube [34], and GABA_C_ receptors have been reported in gut [35], testis and spermatozoa [36].

The fact that human corneal stem cells express GABA_A_ receptors [5], that there are reports of some GABA actions in the cornea of animal species [16,17], that GABA receptors are present in a range of non-neuronal tissues [21-36], and that the chick sclera possess GABA_C_ receptors [15], lead to the hypothesis that GABA receptors may be present in chick cornea. The aim of this study was to determine if GABA_A_, GABA_B_, and/or GABA_C_ receptors are located in the chick cornea. The secondary aim was to determine if expression occurred in the corneal endothelium, stroma or epithelium.

## Methods

This research comprised: (1) study of the expression of alpha_1_ GABA_A_, GABA_B_, and rho_1_ GABA_C_ receptors mRNA in chick cornea, and (2) identification and distribution of alpha_1_ GABA_A_, GABA_B_, and rho_1_ GABA_C_ receptors protein in chick cornea.

### Animals and tissue preparation

Animals and tissue were prepared as previously described [15]. Twenty-five 12-day-old White Leghorn cockerels (*Gallus gallus*) were obtained from Jinan Spafury Poultry Farms (Jinan, China). Animals were administered a lethal dose of pentobarbital sodium (3%, 5ml/kg; Beijing Chemistry Com., Beijing, China) and the eyes enucleated. Eyes (n=5, 1 eye from 5 chicks) for immunohistochemistry were placed whole into microtubes, snap frozen in liquid nitrogen and stored at −80 °C. Eyes for real time PCR (n=5 different samples, 4 eyes of 2 chicks were used as 1 sample, 20 eyes of 10 chicks were used) and western Blot (n=5 different samples, 4 eyes of 2 chicks were used as 1 sample, 20 eyes of 10 chicks were used) were placed on a cold plate (8 °C) and the corneal tissue was removed along the limbus, and was separated from the sclera, conjunctiva, and iris using surgical scissors and forceps under a dissection scope. Corneal tissues from 4 eyes of 2 chicks formed one sample and were placed in separate microtubes, snap frozen in liquid nitrogen and stored at −80 °C until processing. Retina from these eyes were obtained and used as the positive control; the retina has been previously shown to possess alpha_1_ GABA_A_ [6-8], GABA_B_ [9-11], and rho_1_ GABA_C_ [12] receptors.

Experiments were conducted with ethics approval in accordance with the Australian Code of Practice for the Care and Use of Animals for Scientific Purposes, published by the National Health and Medical Research Council of Australia. All animal care and experimental protocols complied with the Animal Management Rules of the Ministry of Health of the People's Republic of China (document No 55, 2001).

### Real-time PCR

Real-time PCR was performed as previously described [15,37] with minor modification according to the manufacturer’s instructions. Total RNA was extracted from chick cornea and retina using Trizol Reagent (Invitrogen, Carlsbad, CA). RNA concentration and purity were determined at an optical density ratio of 260:280 using a spectrophotometer. CDNAs (cDNAs) were synthesized with 1 μg of total RNA, 1 μl random primer,1 μl dNTPs, 2 μl DTT, and 200 U MMLV reverse transcriptase, 5× RT buffer (4 μl) at 37 °C for 50 min, followed by 70 °C for 15 min, using a TaqMan Reverse-Transcription kit from Invitrogen.

Samples were analyzed in triplicate using gene-specific chicken primers together with SYBR Green (TaKaRa Biotechnology Co. Ltd., Dalian, China) using a Real-time PCR Detection System, LightCycler (Roche Applied Science, Indianapolis, IN). Based on the sequences reported in the GenBank database, primers were selected from chick sequences of alpha_1_ GABA_A_ (NCBI Reference Sequence: NM_204318.2), GABA_B_ (NCBI Reference Sequence: XM_419066.3) and rho_1_ GABA_C_ (NCBI Reference Sequence: XM_426190.2) receptors, using NCBI primer-BLAST, targeting at areas non-homologous to the other mRNA sequence, and ordered from Shanghai Biosune Biotechnology Company (Shanghai, China). The sequences of alpha_1_ GABA_A_ were 5′-CTC CCT AAG GTG GCC TAC GCC-3′ forward and 5′-AAT GGT TGC CAG CCC AGG GTC-3′ reverse. The sequences of GABA_B_ were 5′-TCG GGA CCA ACC CAA CGT GC-3′ forward and 5′-CGT GCT GGC CTG ATT GAC GCT-3′ reverse. The sequences of rho_1_ GABA_C_ were 5′-TCG GTG CTG GAA TAC GCG GC-3′ forward and 5′-GGG CTG AGG AAG GCT GCA CG-3′ reverse.

A typical reaction was performed in 20 μl, consisting of 1 μl of cDNA and 10 μl of 2× SYBR Green I PCR mix, containing the specific primer pairs (final 10 pmol each). Denaturation was performed for 10 s at 95.0 °C, primer annealing for 10 s at 60 °C, and extension was performed for 10 s at 72.0 °C. Correct product size was confirmed by DNA agarose gel, and lack of primer dimer formation was verified by melt curve analysis, and the real time PCR products were sent to Shanghai Biosune Biotechnology Company (Shanghai, China) for sequence analysis. For real time PCR, comparing to the cornea, the samples with cDNA from the retina were used as the positive control, and the samples without cDNA were used as the negative control. Samples were analyzed in triplicate.

### Western Blot

Western blot was performed as previously described [15]. For rho_1_ GABA_C_, we used the antibody as previously described [15]. For alpha_1_ GABA_A_ and GABA_B_, we used the goat anti-human alpha_1_ GABA_A_ and GABA_B_ receptor polyclonal antibody (Santa Cruz Biotechnology, Santa Cruz, CA). The homology for the antibody sequence between chicken and human was more than 90% for the alpha_1_ GABA_A_ (Homologene Blast comparison of human alpha_1_ GABA_A_, accession number: NP_001121120.1, with *Gallus gallus* alpha_1_ GABA_A_ receptor, accession Number, P19150.1), and 100% for the GABA_B_ (Homologene Blast comparison of human GABA_B_, accession number: NP_005449.5, with *Gallus gallus* GABA_B_ receptor, accession Number, XP_419066.3).

Total protein was extracted separately from each tissue sample by lysing the cornea and retina in ice-cold lysis buffer (Beyotime Institute of Biotechnology, Shanghai, China), including 50 mM Tris (pH 7.4), 150 mM NaCl, 1% Triton X-100, 1% sodium deoxycholate, and 0.1% SDS. Samples were centrifuged at 15,000× g at 4 °C for 15 min. The protein concentration was detected using BAC kits (Beyotime Institute of Biotechnology). Aliquots of protein extracts were loaded in each lane of 7.5% sodium dodecyl sulfate-polyacrylamide gels, transferred onto polyvinylidene difluoride membranes for electrophoresis, and blocked in Tris Buffered Saline with Tween (TBST; 5% fat-free dry milk, 0.1% Tween-20, 150 mM NaCl, and 50 mM Tris at pH 7.5) for 2 h. The membranes were exposed to goat anti-human alpha_1_ GABA_A_, GABA_B_, and rho_1_ GABA_C_ polyclonal antibody (Santa Cruz Biotechnology) at a 1:100 dilution in blocking buffer and incubated overnight at 4 °C. This was followed by incubation with a rabbit anti-goat secondary horseradish peroxidase (HRP)-labeled antibody (Zhongshan Goldenbridge Biotechnology Co. LtD. Beijing, China) at a dilution of 1:5,000 for 1 h at 37 °C. Protein bands were exposed to a negative film, developed, and fixed. The film was scanned and analyzed with Fluorchem^TM^ 9900 Analyzer Software. β-actin (Zhongshan Goldenbridge Biotechnology Co. LtD.) was used as a housekeeping protein to normalize the protein load. Samples were analyzed in triplicate.

### Immunohistochemistry

Immunohistochemistry was used to investigate the expression and distribution of alpha_1_ GABA_A_, GABA_B_, and rho_1_ GABA_C_ receptors in chick cornea at the protein level, and was performed as has been described previously [15,38]. Whole eyes were freeze-mounted onto sectioning blocks. Vertical sections (8 μm thick) were cut from the anterior pole of the eye on a Leica RM2125 microtome (Leica Microsystems, Muenster, Germany) and thaw-mounted onto gelatin-coated glass slides. Sections were fixed for 10 min in acetone. Fixed sections were washed three times with PBS, covered with 10% BSA (Sigma, St Louis, MO) diluted in PBS, and incubated for 20 min at 37 °C. The slides were incubated at 4 °C overnight with primary antibodies (goat anti-human alpha_1_ GABA_A_, GABA_B_, and rho_1_ GABA_C_ receptors polyclonal antibody, Santa Cruz Biotechnology) at a 1:50 dilution in blocking buffer. Sections were incubated in PBS without primary antibodies as a negative control. The antibody-treated and negative control sample slides were washed with PBS and exposed to a rabbit anti-goat secondary horseradish peroxidase (HRP)-labeled antibody (Zhongshan Goldenbridge Biotechnology Co. LtD.) at a dilution of 1:500 for 1 h at 37 °C. The slides were washed in PBS three times. A nickel solution of DAB (3,3′-diaminobenzidine tetrahydrochloride; Zhongshan Goldenbridge Biotechnology Co. LtD.) was prepared and applied to the sections for less than 30 s. The DAB was then thoroughly rinsed from the sections using water. Sections were then stained with Hematoxylin and Eosin. The sections were dehydrated three times for 1 min in 100% ethanol, and then cleared in Histoclear (Huntz Biotechnology Co. LtD. Shanghai, China) three times for 1 min. The sections were examined with a light microscope (40×) and the images were digitized using a camera. Samples were analyzed in triplicate.

## Results

### Alpha_1_ GABA_A_, GABA_B_, and rho_1_ GABA_C_ receptor mRNA expression in chick cornea

**Figure 1:**
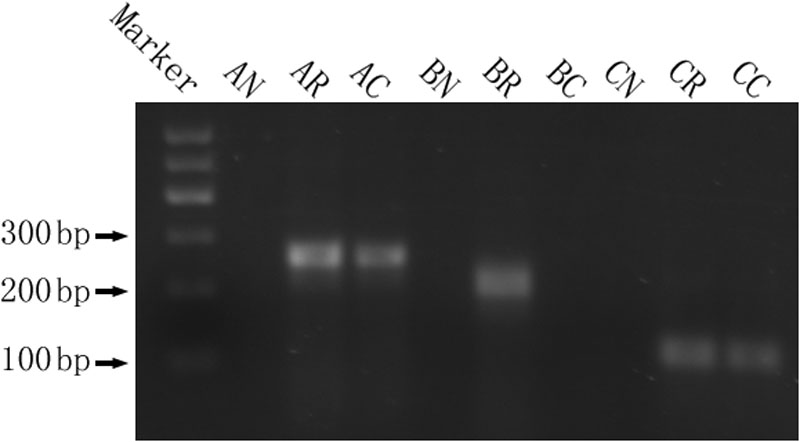
Sample ethidium bromide gel of real-time PCR products of alpha_1_ GABA_A_, GABA_B_ and rho_1_ GABA_C_ receptors in chick cornea and retina. Lane 1: molecular weight ladder (Marker), Lane 2: negative control for alpha_1_ GABA_A_ (AN), Lane 3: chick retina for alpha_1_ GABA_A_ (AR), Lane 4: chick cornea for alpha_1_ GABA_A_ (AC), Lane 5: negative control for GABA_B_ (BN), Lane 6: chick retina for GABA_B_ (BR), Lane 7: chick cornea for GABA_B_ (BC), Lane 8: negative control for rho_1_ GABA_C_ (CN), Lane 9: chick retina for rho_1_ GABA_C_ (CR), Lane 10: chick cornea for rho_1_ GABA_C_ (CC).

We observed the mRNA expression of alpha_1_ GABA_A_ and rho_1_ GABA_C_ receptors in all chick cornea and retina, but not in the negative control. We observed the mRNA expression of GABA_B_ receptors only in chick retina, but not in the cornea and not in the negative control. Ethidium bromide-stained agarose gels of real time PCR products were positive for mRNA expression of alpha_1_ GABA_A_ and rho_1_ GABA_C_ receptors in chick cornea and retina, but not in the negative control run, and for mRNA expression of GABA_B_ receptors only in samples of chick retina, but not in the cornea and negative control run. Products corresponding to alpha_1_ GABA_A_, GABA_B_, and rho_1_ GABA_C_ receptors were amplified and expression occurred at the nucleic acid size marker base pair consistent with that of the alpha_1_ GABA_A_ (255 bp), GABA_B_ (217 bp), and rho_1_ GABA_C_ (105 bp) receptors (Figure 1). The sequence analysis of the real time PCR products revealed that the sequence of the products corresponded to the targeted sequence of the mRNA of the alpha_1_ GABA_A_, GABA_B_, and rho_1_ GABA_C_ receptors with the primers.

### Alpha_1_ GABA_A_, GABA_B_, and rho_1_ GABA_C_ receptor protein expression in chick cornea

**Figure 2:**
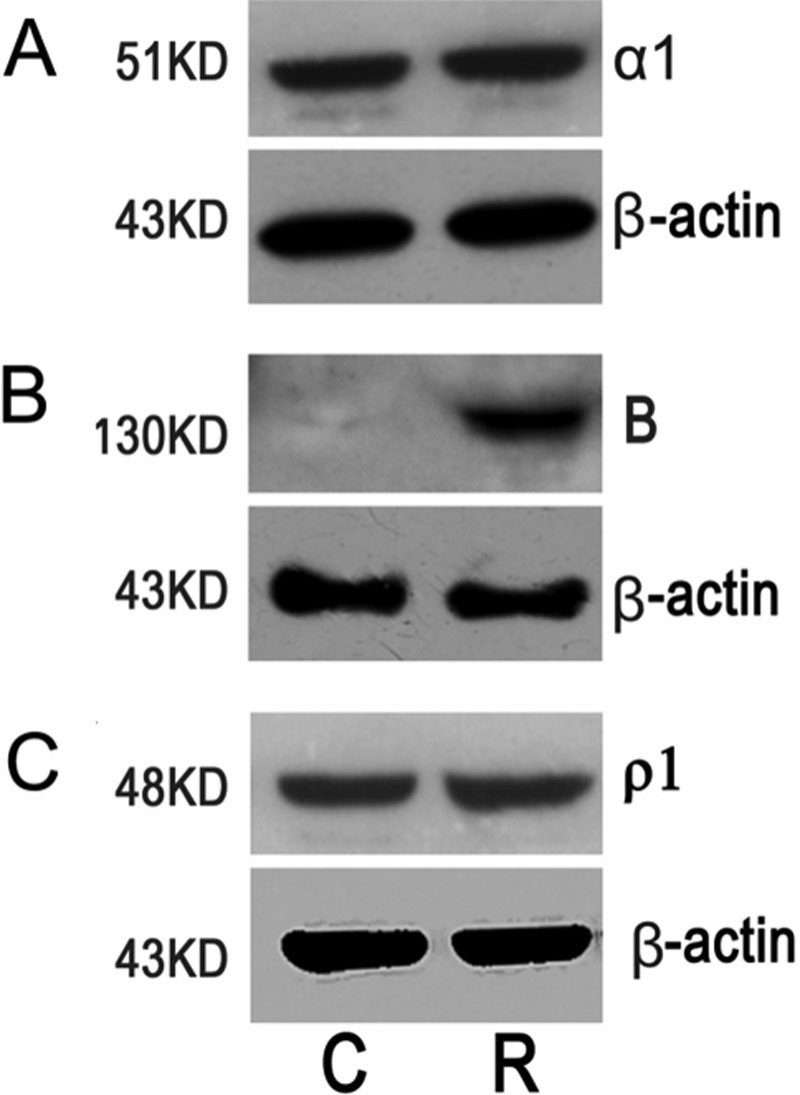
Western blot analysis of alpha_1_ GABA_A_, GABA_B_ and rho_1_ GABA_C_ receptors in the chick cornea and retina. **A**: This panel shows one intense band (approximately 43 kDa) for β-actin and one intense band (approximately 51 kDa) for alpha_1_ GABA_A_ receptors (α1) both in cornea (C) and in retina (R). **B**: This panel shows one intense band (approximately 43 kDa) for β-actin both in cornea (C) and in retina (R), and one intense band (approximately 130 kDa) for GABA_B_ receptors (B) in retina (R) but not in cornea (C). **C**: This panel shows one intense band (approximately 43 kDa) for β-actin, and one intense band (approximately 48 kDa) for rho_1_ GABA_C_ receptors (ρ1) both in cornea (C) and in retina (R).

Using antibodies for alpha_1_ GABA_A_ receptors, one intense band (approximately 51 kDa) was detected in all chick corneal and retinal samples. Using antibodies for GABA_B_ receptors, one intense band (approximately 130 kDa) was detected in the chick retina samples but was not present in the corneal samples. Using antibodies for rho_1_ GABA_C_ receptors, one intense band (approximately 48 kDa) was detected in the chick corneal and retinal samples. Using antibodies for β-actin, one intense band (approximately 43 kDa) was detected in all the chick corneal and retinal samples (Figure 2).

### Alpha_1_ GABA_A_, GABA_B_, and rho_1_ GABA_C_ receptor localization in chick cornea

**Figure 3:**
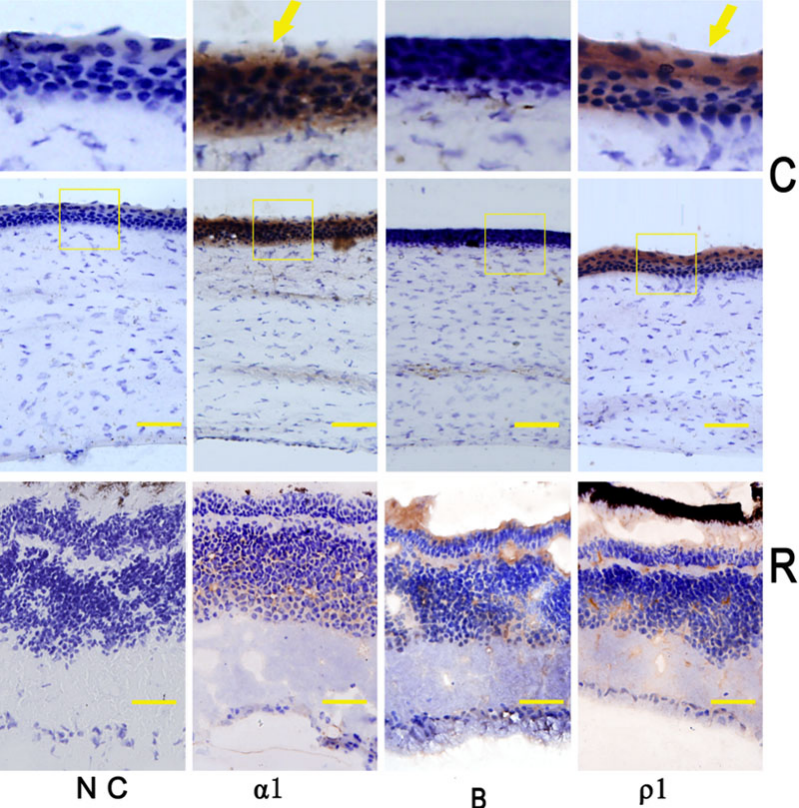
Alpha_1_ GABA_A_ (α1), GABA_B_ (B) and rho_1_ GABA_C_ (ρ1) receptor protein distribution in chick cornea (C) and retina (R). With antibodies for alpha_1_ GABA_A_, GABA_B_ and rho_1_ GABA_C_ receptors, immunoreactivity was observed in corneal epithelium cell for alpha_1_ GABA_A_ (α1) and rho_1_ GABA_C_ (ρ1; yellow arrow), but not for GABA_B_ receptor (B) in the cornea (C). In the retina (R), immunoreactivity was found in the inner plexiform layer, outer plexiform layer, inner nuclear layer, and ganglion cell layer for alpha_1_ GABA_A_ (α1), GABA_B_ (B) and rho_1_ GABA_C_ (ρ1) receptors, corresponding to previous reports [6-11,15]. There was essentially no immunoreactivity observed for the negative controls (N.C). The scale bar is 10 µm. Photographs were taken at 40× magnification.

Alpha_1_ GABA_A_, and rho_1_ GABA_C_ immunoreactivity was observed in chick corneal epithelium, was not observed in the stroma and not in the endothelium. No corneal layer displayed immunoreactivity to GABA_B_ antibodies. In the retina, immunoreactivity for each of the antibodies was observed in the inner plexiform layer, outer plexiform layer, inner nuclear layer and ganglion cell layer, corresponding with previous published data [6-11,15]. There was essentially no immunoreactivity observed in the negative controls tissues (Figure 3).

## Discussion

Our results add new information on the distribution of GABA receptors within ocular tissues; GABA_A_ receptors have been previously reported to be localized to a few cultured human corneal stem cells [5], here we clearly show that the chick cornea is positive for mRNA and protein specific for alpha_1_ GABA_A_ and rho_1_ GABA_C_ receptors, but negative for GABA_B_ receptors. It would be of great interest to know if the corneas of other animal species also express GABA receptors and if so which sub-receptor types and in which corneal tissues. Staining density suggests that the alpha_1_ GABA_A_ and rho_1_ GABA_C_ receptors occur throughout the entire chick corneal epithelial layer, and are located on epithelial cells. The finding suggests a possible, but untested, role of the alpha_1_ GABA_A_ and rho_1_ GABA_C_ receptors in control of corneal functions.

Receptor types that are generally thought of as having primarily retinal localizations and function have been observed in corneal tissue. This includes muscarinic receptors [2,19,20,37], dopamine receptors [4], melatonin receptors [3], and retinoic acid receptors [18], here we show that the alpha_1_ GABA_A_ and rho_1_ GABA_C_ receptors, but not GABA_B_ receptors are located in chick corneal epithelium. The functions mediated through these different receptors are diverse, eg sensation and epithelial cell proliferation (muscarinic receptors [19,20]), ion transport (dopamine receptors [39]), diurnal variations in corneal hydration and thickness (melatonin [40]), and maintenance of the ocular surface (retinoic acid receptors [18]).

Muscarinic receptor subtypes have been observed on bovine corneal epithelial cells [2], and on human corneal epithelium and endothelium [19,20]. The muscarinic receptor agonist, carbachol (0.001–100 microM) can increase the intracellular Ca^2+^ concentration in bovine corneal epithelial cells, and if the cells were preincubated with either 1 microM atropine or 1 microM pirenzipine [2] this was suppressed. When rats were fed a muscarinic agonist, corneal opacities with histopathological features including neovascularization, acanthosis, and stromal proliferation were observed in a dose-related fashion at 100 and 200 mg/kg/day [41]. Dopamine receptors, the D2 subtype, are located in rabbit cornea epithelial and endothelial layers [4]. Dopamine increase Cl^-^ secretion by the activation of specific dopamine receptors, and stimulate ion transport within the rabbit corneal epithelium [39]. Melatonin receptors (Mel_1a_ [40]) are expressed in chick corneal epithelium, stroma and endothelium. It has been suggested [40] that melatonin may modulate daily rhythms in corneal hydration/thickness via melatonin receptors on the corneal endothelium. Unlike dopamine, muscarinic and melatonin receptors that are localized in both epithelium and endothelium, here alpha_1_ GABA_A_ and rho_1_ GABA_C_ are only located in epithelium.

One of the important functions of the cornea is maintaining its optical transparency which is crucial for high quality visual performance. Corneal transparency is dependent on regulation of the hydration of the cornea, and the Cl^-^ ion channel is involved in fluid transportation within the corneal epithelium and endothelium [42-44]. The presence of Cl^-^ ion channels has been reported in human and rabbit corneal epithelium [45,46], in rabbit endothelium [45], and in rabbit corneal keratocytes [47,48]. GABA and its analogs have been shown to activate the rabbit corneal endothelial fluid pump, and this stimulation was abolished by the GABA_A_ antagonists, bicuculline and chlorpromazine [16]. In this study we found alpha_1_ GABA_A_ and rho_1_ GABA_C_ receptors in chick corneal epithelium, but not the endothelium, and thus it seems unlikely that GABA is involved in regulating the fluid pumps, at least within the chick corneal endothelium. Whether alpha_1_ GABA_A_ and rho_1_ GABA_C_ receptors regulate Cl^-^ conductance, and then influence transparency within the chick cornea requires further investigation. Determination of the effect of GABA agents on corneal hydration would resolve this.

To maintain corneal composition, organization and clarity the cornea has the greatest density of peripheral sensory nerves of any ocular tissue. Nerve terminals in the cornea are almost exclusively nociceptive Aδ and C fibers originating from the ophthalmic branch of the trigeminal ganglion [49], and structural and functional specialization of Aδ and C fiber free nerve endings innervate the corneal epithelium [50] across the entire corneal surface [1]. In this study we found alpha_1_ GABA_A_ and rho_1_ GABA_C_ receptor within the chick corneal epithelium. A potential role for these GABA receptors might involve regulating corneal sensitivity or other neural functions and this requires further investigation.

If the GABA receptors are to have a functional role within the cornea then there must be a supply of GABA either within the cornea, aqueous humor or tears to interact with the receptors. GABA-synthesizing enzyme was observed in the rat cornea [17], which implys that there is endogenous synthesis of GABA in rat cornea. It may be possible that cholinergic neurons within the cornea release GABA, like they do within retinal tissue [51]. We could not locate any articles that mention the presence of GABA within the eye’s aqueous humor or tears. All of these potential sources of GABA are yet to be tested in the chick eye.

In conclusion, we found that both alpha_1_ GABA_A_ and rho_1_ GABA_C_ receptor were located within the chick corneal epithelium; the GABA_B_ receptor was not present. The presence of GABA receptors within chick corneal epithelium suggests that some epithelial cell functions can be modified by GABA. Research is underway to determine how activation of these receptors alters corneal functions and where GABA or other substances that modifies them are expressed.
